# Treatment of *Aspergillus fumigatus* in Patients with Cystic Fibrosis: A Randomized, Placebo-Controlled Pilot Study

**DOI:** 10.1371/journal.pone.0036077

**Published:** 2012-04-30

**Authors:** Shawn D. Aaron, Katherine L. Vandemheen, Andreas Freitag, Linda Pedder, William Cameron, Annick Lavoie, Nigel Paterson, Pearce Wilcox, Harvey Rabin, Elizabeth Tullis, Nancy Morrison, Felix Ratjen

**Affiliations:** 1 The Ottawa Hospital Research Institute, University of Ottawa, Ottawa, Ontario, Canada; 2 McMaster University, Hamilton, Ontario, Canada; 3 Centre Hospitalier de L'Universite de Montreal, Montreal, Quebec, Canada; 4 University of Western Ontario, London, Ontario, Canada; 5 University of British Columbia, Vancouver, British Columbia, Canada; 6 University of Calgary, Calgary, Alberta, Canada; 7 St. Michael's Hospital, University of Toronto, Toronto, Ontario, Canada; 8 Capital Health District, Halifax, Nova Scotia, Canada; 9 Hospital For Sick Children, University of Toronto, Toronto, Ontario, Canada; Postgraduate Institute of Medical Education and Research, India

## Abstract

**Background:**

Many patients with cystic fibrosis develop persistent airway infection/colonization with *Aspergillus fumigatus*, however the impact of *A. fumigatus* on clinical outcomes remains unclear. The objective of this study was to determine whether treatment directed against *Aspergillus fumigatus* improves pulmonary function and clinical outcomes in patients with cystic fibrosis (CF).

**Methods:**

We performed a double-blind randomized placebo-controlled pilot clinical trial involving 35 patients with CF whose sputum cultures were chronically positive for *A. fumigatus*. Participants were centrally randomized to receive either oral itraconazole 5 mg/kg/d (N = 18) or placebo (N = 17) for 24 weeks. The primary outcome was the proportion of patients who experienced a respiratory exacerbation requiring intravenous antibiotics over the 24 week treatment period. Secondary outcomes included changes in FEV_1_ and quality of life.

**Results:**

Over the 24 week treatment period, 4 of 18 (22%) patients randomized to itraconazole experienced a respiratory exacerbation requiring intravenous antibiotics, compared to 5 of 16 (31%) placebo treated patients, P = 0.70. FEV_1_ declined by 4.62% over 24 weeks in the patients randomized to itraconazole, compared to a 0.32% improvement in the placebo group (between group difference = −4.94%, 95% CI: −15.33 to 5.45, P = 0.34). Quality of life did not differ between the 2 treatment groups throughout the study. Therapeutic itraconazole blood levels were not achieved in 43% of patients randomized to itraconazole.

**Conclusion:**

We did not identify clinical benefit from itraconazole treatment for CF patients whose sputum was chronically colonized with *A. fumigatus*. Limitations of this pilot study were its small sample size, and failure to achieve therapeutic levels of itraconazole in many patients.

**Trial Registration:**

ClinicalTrials.gov NCT00528190

## Introduction

*Aspergillus fumigatus* is a common fungal organism found in the sputum of patients with cystic fibrosis (CF) [1], [2]. Evidence from observational studies suggests that *A. fumigatus* is more commonly isolated from older CF patients and from those who use chronic maintenance therapy with inhaled antibiotics [3], [4]. Whereas the pathophysiological role of major bacteria, such as *Pseudomonas aeruginosa*, is well established in CF, the relevance of fungi is poorly understood [5], [6].

National registry studies indicate that prevalence of *A. fumigatus* infection is increasing in the CF population. In Canadian patients with CF the prevalence of *A. fumigatus* isolated from sputum rose from 8% in 2001 to 18% in 2009 [7], [8]. As the CF population ages, and as intensive antibiotic suppressive therapy for bacterial infection becomes more common, the incidence and prevalence of *A. fumigatus* infection is increasing.

A subgroup of CF patients who are colonized with *A. fumigatus* develop allergic bronchopulmonary aspergillosis (ABPA), an immune-mediated hypersensitivity to aspergillus that is manifested with wheezing and declining lung function [9]. ABPA is diagnosed based on clinical criteria and evidence of a type I hypersensitivity response against *A. fumigatus* [10]. However, in the absence of ABPA, the impact of persistence of *A fumigatus* in the airways on the course of CF lung disease remains unclear [11].

A retrospective cohort study from the pediatric CF clinic in Toronto revealed that CF patients persistently infected with *A. fumigatus*, who did not have ABPA, had a lower FEV_1_ compared to uninfected patients, and these patients were also at a higher risk for pulmonary exacerbations requiring hospitalization (RR = 1.94, P = 0.0002) [12]. Similarly, a 3-year prospective cohort study of adult CF patients from Canada revealed that the prevalence of chronic infection with *A. fumigatus* was twice as high in patients with frequent CF exacerbations compared to those with infrequent exacerbations [13]. Finally, a recent case series from Israel described a group of 6 CF patients with sputum cultures positive for *A. fumigatus* who presented with respiratory deterioration that did not respond to antibiotic treatment [14]. Treatment with the antifungal agent itraconazole for 4–24 months resulted in significant improvement in the patients' clinical conditions. The authors suggested that aspergillus-related bronchitis may be an over-looked and largely untreated disease in CF patients [14].

The current standard of care amongst CF centers is to forego antifungal treatment in CF patients who culture *A. fumigatus* in sputum but who do not have ABPA [10]. However to date, no prospective experimental studies have addressed the question of whether treating aspergillus in patients with cystic fibrosis will improve clinical outcomes. We therefore conducted a randomized, placebo-controlled pilot clinical trial to determine if 24 weeks of treatment with the oral antifungal agent itraconazole improved clinical outcomes in CF patients whose sputum was chronically colonized with *A. fumigatus*.

## Methods

The protocol for this trial and supporting CONSORT checklist are available as supporting information; see Checklist S1 and Protocol S1.

### Patients

We enrolled patients with CF from 9 Canadian outpatient centers. Patients were included in the study if they were ≥6 years of age and if they had a confirmed diagnosis of cystic fibrosis made via genetic analysis and/or sweat testing. Eligible patients had to be chronically colonized with *Aspergillus fumigatus*; defined as at least 2 positive sputum cultures within the last 12 months, one of which had to be obtained within 4 months of randomization. Patients were excluded if they had a history of renal insufficiency (serum Cr >1.5 times normal), significant liver disease (defined as serum AST or ALT≥2.5 times the upper limit of normal), or a history of biliary cirrhosis or portal hypertension. Patients were also excluded if they had active allergic bronchopulmonary aspergillosis (ABPA), *B. cepacia* infection, lung transplantation, or if they had received treatment with any antifungal agents within 6 months before randomization. Patients were required to be clinically stable at the time of randomization, with no antibiotic treatment for acute CF pulmonary exacerbations allowed for at least 14 days prior to randomization.

### Study Design

The study was a double-blind, randomized, placebo-controlled, multi-centre, pilot clinical trial incorporating two parallel treatment arms. Patients underwent study assessments at baseline, and at 4, 12, 24, and 48 weeks after randomization.

### Ethics

The Research Ethics Boards of all of the participating hospitals approved the study; project approval numbers from each Research Ethics Board are listed in brackets: The Ottawa Hospital (2006-768), The Hospital for Sick Children's Hospital (1000011289), St. Michael's Hospital (07-242), Conjoint Health (E-21687), Hamilton Health Sciences (08-216), University of British Columbia Providence Healthcare (H08-0204), Centre Hospitalier de L'Universite de Montreal (08-190), The University of Western Ontario Hospitals (15843), Queen's University Health Sciences and Affiliated Hospitals (DMED-1178-08), and Capital Health Hospitals (CHDA-RS-2009-283). All patients (and their parents when applicable) signed informed consent prior to study entry.

### Study intervention

Patients were randomly allocated to either daily oral itraconazole capsules or identical placebo capsules for a 24 week treatment period. Dosing of itraconazole was calculated to provide a daily dose of 5 mg/kg/d as per CF Consensus Guidelines [11]. Itraconazole, or identical placebo, was given once daily by mouth, unless the dose exceeded 200 mg/day, in which case it was given twice daily. Patients were advised to take study medication with orange juice or at least 8 oz of a cola beverage in order to maximize oral absorption. Patients were also instructed to take the study medication at least 4 hours before using medications which decrease stomach acidity, such as histamine-blockers or proton pump inhibitors. All study patients otherwise continued standard therapy for their CF as prescribed by their treating physician.

### Randomization

A central allocation schedule for randomisation was prepared through a computer-generated random listing of the two treatment allocations in variable blocks of two or four. Study medication was dispensed by the site research pharmacist according to the patient's randomization assignment. Research staff and medical staff were unaware of the treatment assignment before or after randomization.

### Outcome measures

The primary endpoint for this study was the proportion of patients who experienced a respiratory exacerbation requiring intravenous antibiotics over the 24 week trial treatment period. The original intention was to extend the pilot into a larger definitive study of 328 patients; such a study would have been adequately powered to assess exacerbation rates as the primary outcome. Extension of the pilot study into the larger definitive study was dependent on demonstration of feasibility of recruitment, and was dependent on additional funding being obtained for the larger, definitive study. Neither of the latter two conditions was met, and the pilot study was therefore not extended.

Secondary outcomes included the percentage change in FEV_1_ from baseline over the 24 week treatment period, as well as time to first exacerbation and the number of exacerbations requiring intravenous or oral antibiotics/patient-year. Changes in disease-specific health-related quality of life over the 24 week study treatment were assessed by the Cystic Fibrosis Questionnaire (CFQ-R)- a disease-specific instrument that measures health-related quality of life for children, adolescents and for adults with cystic fibrosis [15]. CFQ-R scores were standardized on a 0 to 100 point scale with higher scores representing better quality of life. Incidence of adverse effects and serious adverse events were also assessed. All efficacy and safety outcomes listed were assessed at 24 weeks, but also at 48 weeks after randomization, to determine if there were any lasting positive or negative carry-over effects of 24 weeks of therapy with itraconazole.

### Statistical analysis

This was a pilot study to determine feasibility of a larger clinical trial, and a convenience sample size of 60 was selected for the pilot phase. Recruited patients were followed for 48 weeks.

Changes in FEV_1_ from baseline to week 24, and to week 48, were compared in the 2 treatment groups using Student's t-test. We also compared change in FEV_1_ using linear regression analysis adjusting for patient age, gender and baseline FEV_1_ % predicted. The proportion of patients in the 2 groups who experienced respiratory exacerbations was analyzed using Chi-square tests. Kaplan Meir survival curves were used to compare time from randomization to first exacerbation for the 2 groups. Cox proportional hazards models were also used to adjust for patient age, gender and baseline FEV_1_% predicted. The mean number of exacerbation per patient-week was calculated using a weighted approach to account for each patient's duration of follow-up in the trial. A rate ratio was produced to compare the number of exacerbations/pt-week in the 2 groups. The 95% CI of the rate ratio was obtained using a time-weighted Poisson regression analysis with an incorporated overdispersion parameter using the SAS GENMOD procedure. All analyses were intention to treat and were conducted using SAS software, version 9.0 (SAS Institute, Inc., Cary, North Carolina).

## Results

### Study Population

Patients were randomized from January 2008 to May 2010. A total of 75 potentially eligible patients were screened for the study however 32 patients declined to participate and 8 were ineligible for other reasons. Recruitment into the study proved to be more difficult than expected, and ultimately the pilot study was halted after 35 patients had been recruited.

**Figure 1:**
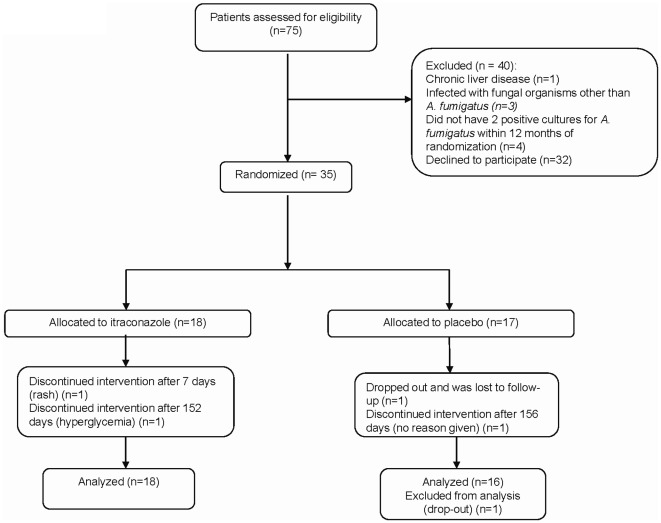
Trial Profile.

Thirty-five patients from 9 Canadian CF clinics were randomized to either receive itraconazole (n = 18) or to receive placebo (n = 17) (Figure 1). One patient randomized to itraconazole developed a rash and had to discontinue the study medication after 7 days, a second patient developed hyperglycemia and discontinued study medications after 152 days. Both patients stayed in the study. One patient in the placebo group stopped study medications prematurely at 156 days but stayed in the study. A second patient in the placebo group dropped out of the study and was lost to follow-up after randomization. No clinical outcome data were available for this patient.

The baseline demographics and clinical characteristics of the two groups are provided in Table 1. No significant differences were observed for any of the baseline clinical characteristics. Baseline serum eosinophil counts, total IgE levels and IgE RAST levels against *A. fumigatus* were not significantly different between the two groups.

**Table 1:** Baseline Characteristics of the Randomized Patients.

	Placebo (N = 17)	Itraconazole (N = 18)
Mean Age (SD)	25.2 (9.1)	25.3 (10.5)
Male (%)	9 (53%)	10 (56%)
BMI (kg/m^2^) (SD)	21.4 (4.2)	21.1 (3.1)
FEV_1_ (L) (SD)	2.31 (0.90)	2.09 (0.75)
FEV_1_% predicted (SD)	64.9% (22.2)	63.4% (18.1)
FVC% predicted (SD)	82.9% (17.4)	80.8% (17.9)
Serum eosinophils (10^9^/L) (SD)	0.15 (0.11)	0.17 (0.14)
Serum IgE level (ug/L) (SD)	159 (303)	247 (335)
Serum IgE specific RAST against *A*. *fumigatus* (KU/L) (SD)	1.18 (2.81)	1.69 (4.05)
Comorbidities:		
Diabetes	3 (18%)	5 (28%)
Pancreatic insufficiency	14 (82%)	16 (89%)
Coinfections:		
*S. aureus*	8 (47%)	7 (39%)
*P. aeruginosa*	10 (59%)	7 (39%)
*S. maltophilia*	2 (12%)	7 (39%)
Medications:		
Azithromycin	7 (41%)	10 (56%)
Inhaled tobramycin	12 (71%)	10 (56%)
Dornase alpha	8 (47%)	4 (22%)
Inhaled hypertonic saline	6 (35%)	4 (22%)
Inhaled corticosteroids	12 (71%)	12 (67%)

### Primary outcome

The primary outcome was the proportion of patients who experienced a respiratory exacerbation requiring intravenous antibiotics over the 24 week treatment period (Table 2). Over the 24 week treatment period, 4 of 18 (22%) patients randomized to itraconazole experienced a respiratory exacerbation requiring intravenous antibiotics, compared to 5 of 16 (31%) placebo treated patients, P = 0.70.

**Table 2:** Pulmonary Exacerbations and Hospitalizations by Treatment Group - 24 Week Treatment Period.

24-Week Treatment Period	Placebo (N = 16)	Itraconazole (N = 18)	P- value
Exacerbations requiring intravenous antibiotics	5 (31%)	4 (22%)	0.70
Exacerbations requiring oral or intravenous antibiotics	7 (44%)	12 (67%)	0.18
Hospitalizations	3 (19%)	3 (17%)	0.99
Pulmonary exacerbations/patient-year	1.59	1.87	Rate Ratio = 1.18 (95% CI 0.55–2.52) P = 0.68

### Secondary outcomes

**Figure 2:**
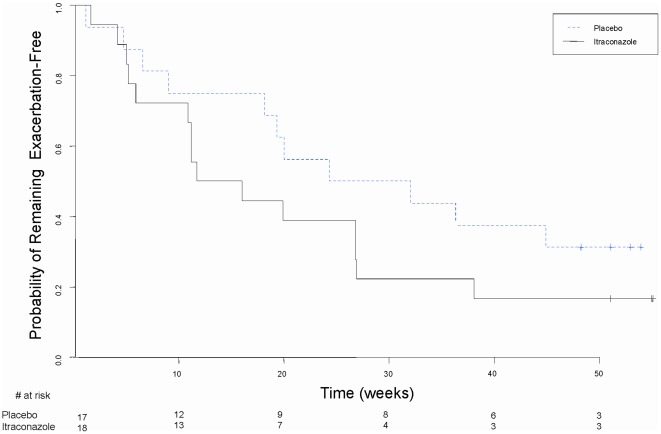
Time to First Pulmonary Exacerbation Requiring Oral or Intravenous Antibiotics. Blue dashed line = placebo-treated patients. Black solid line = itraconazole-treated patients. The median time to first exacerbation was 77 days for the itraconazole group and 134 days for the placebo group, log-rank P = 0.35. Hash marks = censored observations.

The time to first exacerbation requiring oral or intravenous antibiotics was not significantly different between groups (median time to first exacerbation = 77 days for the itraconazole group and 134 days for the placebo group, log-rank P = 0.35) (Figure 2). Adjustment of the time to first exacerbation for age, gender and baseline FEV_1_ did not change the results (hazard ratio = 1.34; 95% CI: 0.57–3.14, P = 0.50).

Over the 24 week treatment period 12 of 18 patients (67%) randomized to itraconazole experienced at least one pulmonary exacerbation requiring intravenous or oral antibiotics, compared to 7 of 16 (44%) evaluable patients randomized to placebo (P = 0.18) (Table 2). Patients in the itraconazole-treated group and the placebo-treated groups experienced a mean of 1.87 exacerbations/patient-year (0.036 exacerbations/patient-week) and 1.59 exacerbations/patient-year (0.031 exacerbations/patient-week) respectively requiring oral or IV antibiotics, rate ratio = 1.18 (95% CI: 0.55–2.52, P = 0.68) (Table 2). Similarly, there were no significant changes in exacerbation rates or hospitalizations between the two groups when assessed over the 48 week follow-up period (Table 3).

**Table 3:** Pulmonary Exacerbations and Hospitalizations by Treatment Group - 48 Week Observation Period.

48-Week Observation Period	Placebo (N = 16)	Itraconazole (N = 18)	P- value
Exacerbations requiring intravenous antibiotics	5 (31%)	7 (39%)	0.64
Exacerbations requiring oral or intravenous antibiotics	11 (69%)	15 (83%)	0.43
Hospitalizations	3 (19%)	4 (22%)	0.99
Pulmonary exacerbations/patient-year	1.78	2.05	Rate Ratio = 1.15 (95% CI 0.64–2.30) P = 0.55

**Figure 3:**
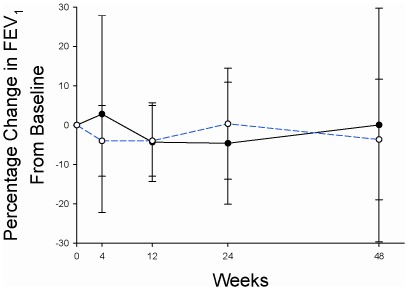
Changes from Baseline in FEV1 Over the 48-Week Study. Blue dashed line = placebo-treated patients. Black solid line = itraconazole-treated patients. 95% confidence intervals are indicated by the error bars around the mean values.

FEV_1_ declined by 4.62% over 24 weeks in the patients randomized to itraconazole, compared to a 0.32% improvement in FEV_1_ in the placebo group (between group difference = −4.94%, 95% CI: −15.33 to 5.45, P = 0.34). Adjustment for age, gender, and baseline FEV_1_% predicted did not change the result, the adjusted between group difference was −4.85%, P = 0.34. Similarly there was no difference in the change in FEV_1_ over the entire 48 week study period (between group difference = 3.71%, 95% CI: −13.26 to 20.68, P = 0.66) (Figure 3).

FVC declined by 1.95% over 24 weeks in the patients randomized to itraconazole, compared to a 1.98% improvement in FVC in the placebo group (between group difference = −3.93%, 95% CI: −14.65 to 6.78, P = 0. 46).

Changes in quality of life were not significantly different in the 2 groups for any of the 12 domains of the CFQ-R. The respiratory domain of the CFQ-R increased by 3.76 points (SD = 10.06) for the itraconazole group and 4.77 points (SD = 24.29) for the placebo group over the 24 week treatment period, the between group difference was 1.01 points, P = 0.87.

### Adverse Effects

Nine of 18 patients randomized to itraconazole reported adverse events compared to 5 of 17 patients in the placebo group (Table 4). There was only one serious adverse event reported in the itraconazole group, a spontaneous pneumothorax that required hospital admission. There were no episodes of hepatitis or elevations in liver enzymes.

**Table 4:** Adverse Events.

Adverse Event	Placebo (N = 16)	Itraconazole (N = 18)
Spontaneous pneumothorax	0	1
Increased dyspnea	2	2
Rash	1	2
Hemoptysis	1	2
Hyperglycemia	0	1
Flu-like illness	0	3
Diarrhea	1	0
Conjunctivitis	1	0

### Plasma Levels of Itraconazole and its Metabolite

Blood was taken from participants 3 and 6 months after randomization to assess plasma levels of itraconazole and its metabolite hydroxy-itraconazole. Plasma was frozen and assays were performed after the trial was completed and the randomization code was broken. Fourteen of 18 patients randomized to itraconazole had plasma assays performed. In six of fourteen patients the measured plasma levels of itraconazole and hydroxyitraconazole were below therapeutic levels of 300 ng/mL, the other 8 patients had levels within the therapeutic range.

## Discussion

This is the first prospective randomized controlled clinical trial to explore whether treatment of chronic *A. fumigatus* airway infection in patients with cystic fibrosis provides clinical benefits. In this study there was no evidence of effect of 6 months of itraconazole treatment in patients with CF who were chronically infected with *A. fumigatus*. Neither lung function, exacerbation rates, nor quality of life differed significantly between the 2 treatment groups after 24 weeks of treatment, nor after a full 48 weeks of observation.

Our original intent was to extend the pilot study into a definitive trial of 328 randomized patients. Unfortunately recruitment into the pilot phase was poor, and the pilot study was not extended into a larger trial. Our study had a relatively high level of patient non-participation (32 of 75 patients assessed for eligibility declined to participate) which may potentially limit the representativeness of our study sample.

Definitive conclusions cannot be drawn from our pilot trial, since the current study is limited by a relative lack of power to show a difference between the 2 treatment groups. However, there was no suggestion of efficacy; patients treated with itraconazole tended to have lower lung function, and tended to have more total exacerbations than those treated with placebo over the 24 week treatment period. Therefore, our pilot study did not show any ‘proof of concept’ results which would support undertaking a larger definitive clinical trial.

There are several explanations, other than lack of statistical power, which can potentially explain why itraconazole did not appear to be clinically effective. The first explanation may be that *A. fumigatus*, although often isolated from the sputum of patients with CF, may not play a pathogenic role in CF patients who do not have allergic bronchopulmonary aspergillosis. If *A. fumigatus* is simply a colonizer of the CF airway, rather than an infecting organism causing tissue injury and inflammation, then treatment of this organism would not be expected to provide clinical benefits. To date, studies in CF patients are inconclusive as to whether *A. fumigatus* is causing infection in these patients [5], [6], [10], and while it cannot be excluded that individual patients may benefit from therapy, this current study would suggest that treatment of this organism on the basis of sputum isolation alone is not indicated in CF patients.

Other potential limitations of our study include eligibility criteria which defined chronic colonization with *Aspergillus fumigatus* as requiring at least 2 positive sputum cultures within the last 12 months, one of which had to be obtained within 4 months of randomization. It is possible that patients' infection status might have changed within the time that elapsed between the last positive sputum culture and randomization. A tighter definition, requiring two positive sputum detections and a positive nucleic acid amplification test, with the last positive culture occurring less than 4 weeks prior to randomization, might have been preferable.

Another potential explanation for lack of efficacy may lie in the variability of pharmacokinetics and absorption of oral itraconazole capsules in CF patients. Although we used a dose of itraconazole of 5 mg/kg/day, which is the dose recommended in CF consensus guidelines for treatment of allergic bronchopulmonary aspergillosis [11], previous studies in CF patients have shown marked inter-subject variability in absorption of oral itraconazole [16], [17]. Itraconazole has the disadvantage of limited oral bioavailability, and the capsule form requires an acidic environment for dissolution which is inhibited by antacid therapies. Liquid forms of itraconazole are better absorbed but are unpalatable. Studies in CF adults have revealed that steady state concentrations are achieved within 8 days of beginning oral itraconazole therapy, but that only 50% of adult CF patients achieve plasma concentrations >250 ng/mL when dosed at 2.5 mg/kg twice daily [16]. Our study suggested similar results, 43% of patients we tested had itraconazole plasma levels below 300 ng/mL, indicating that some patients did not achieve therapeutic itraconazole levels, either because of poor absorption of the drug, or because of non adherence to the treatment protocol. Future trials in patients with CF will need to employ dose titrations of itraconazole based on measurements of serum itraconazole levels.

Aside from problems with absorption and bioavailability, itraconazole is also a first generation azole antifungal agent. Newer second-generation antifungal agents, such as voriconazole and posaconazole, do exhibit a better pharmacokinetic profile, and perhaps better potency against aspergillus species, and it is possible that these newer agents may be more effective [18].

In summary, our pilot randomized controlled trial did not demonstrate any clinical benefits supporting long-term itraconazole treatment for patients with CF whose sputum was chronically positive for *A. fumigatus*. Our study was small, and relatively poor absorption of itraconazole was observed with sub-therapeutic plasma levels in some patients. Future larger trials, using newer second generation azoles, with therapeutic monitoring of drug levels, might be required to definitively determine if long-term antifungal therapy is beneficial for these patients.

## Supporting Information

Checklist S1 **CONSORT Checklist.**
(DOCX)

Protocol S1 **Trial Protocol.**
(DOC)
