# Supplementary material for: Treatment of Aspergillus fumigatus in Patients with Cystic Fibrosis: A Randomized, Placebo-Controlled Pilot Study
Source: PLoS One. 2012 Apr 30;7(4):e36077. doi: 10.1371/journal.pone.0036077 (PMC3340414; doi:10.1371/journal.pone.0036077)
Supplement: Protocol S1 — Trial Protocol. (DOC) [file pone.0036077.s002.doc]

**Protocol S1- Trial Protocol**

**The need for a trial:**

**1.1 What is the problem to be addressed?**

The aim of this study is to determine whether antibiotic treatment directed against *Aspergillus fumigatus* will be effective at preventing respiratory exacerbations and improving pulmonary function in patients with cystic fibrosis (CF) who are chronically colonized/infected with aspergillus. This aim will be accomplished by means of a randomized, double-blind, placebo-controlled clinical trial incorporating two parallel treatment arms.

**1.2 What are the principal research questions to be addressed?**

Primary objective: We wish to determine whether therapy with the oral antifungal antibiotic itraconazole will reduce the absolute rate of CF pulmonary exacerbations over a 24-week treatment period compared to placebo.

Secondary Objectives: To determine whether therapy with itraconazole will improve lung function and health-related quality of life over a 24-week treatment period compared to placebo.

**1.3 Background/ Why is a trial needed now?**

The airways of patients with CF harbor a unique spectrum of bacterial and fungal organisms. *Aspergillus fumigatus* is the most prominent fungal organism found in sputum samples from patients with cystic fibrosis (CF)^1-3^. While studies suggest that approximately 25%, and up to 50%, of CF patients have aspergillus which grows in their sputum, it is still unclear whether the organism is acting as a significant pathogen in CF patients. A small proportion (<5%) of CF patients who culture aspergillus from their sputum develop allergic bronchopulmonary aspergillosis (ABPA), a well established complication of CF lung disease ^4^, however the relevance of aspergillus in the 95% of patients who culture aspergillus from their sputum, but who do not develop allergic bronchopulmonary aspergillosis (ABPA), is unknown.

Currently, most investigators and clinicians consider *A. fumigatus* to be a colonizer of the airways of patients with CF, rather than a pathogen. Therefore the current standard of care is to offer these patients no anti-fungal treatments. Our hypothesis is that *A. fumigatus* is causing disease in these patients, and that the organism is not simply an innocent colonizer of diseased airways. To test this hypothesis we will assess whether the use of a well established intervention against *Aspergillus fumigatus* in chronically infected/colonized patients has a positive effect on clinical outcome parameters (rate of pulmonary exacerbations, pulmonary function, and QOL) in patients with cystic fibrosis.

***Epidemiology and Economic Burden of CF:***

Cystic fibrosis (CF) is a genetic disease caused by abnormalities in the Cystic Fibrosis Transmembrane Regulator (CFTR) protein that controls transepithelial chloride transport in the ciliated epithelium of the conducting airways. CF leads to pathologic changes in organs that express CFTR; therefore secretory cells, sinuses, lungs, pancreas, liver and reproductive tract are involved. Abnormal chloride transport across the respiratory epithelium of patients with CF is associated with increased mucus viscosity in the lungs^1^. The inability of CF patients to clear this thickened mucus can result in chronic, obstructive pulmonary changes, repeated pulmonary infections, and eventually death. Despite increased understanding of the disease, CF remains an incurable life- limiting condition and children and young adults still die prematurely mostly from respiratory insufficiency.

Most patients with CF ultimately succumb to chronic airway infection and inflammation and subsequent respiratory failure. In 1969, CF was primarily a disease of children and median survival in industrialized countries was only 14 years^3^. However today in Canada, CF is a disease of adults and children, 45% of Canadian CF patients are now older than 18, and median survival in Canada is amongst the best in the world and has increased to age thirty-seven^5;6^.

The incidence of CF in Canada and the UK is estimated to be one in 2,500 births^5;6^. Approximately 3500 Canadian patients are registered in the Canadian CF Patient Data Registry and followed in Canadian CF clinics^6^. However despite being an uncommon disease, it’s societal and economic burden are considerable. The disease affects children starting in infancy, and the disease is chronic and kills young and middle-aged adults^7^.

The health-care needs of adults with CF are enormous. Patients with CF require frequent hospitalizations for management of pulmonary infection, and hospitalization rates for patients older than 18 have increased over the past decade^8^. Average direct costs of healthcare services in the US for patients with CF range from $US 6,200 to $US 43,100 per year (calculated in 1996 dollars)^9-12^. A Canadian study of 303 CF patients calculated average direct costs for CF adults of $8,979 per year (figures adjusted to 1996 US dollars)^11^. Inpatient care to treat acute pulmonary exacerbations accounted for 33% of the total direct Canadian healthcare costs^10^. Studies of indirect costs related to incapacity to work, disability, and premature death were estimated at $US 8400 per CF patient per year^11^. In total, actual direct lifetime costs of CF have been estimated at $US 250,000- $US 500,000{55, 4205}. Clearly the economic burden of CF on the healthcare system, and on patients and their caregivers, is considerable.

***Chronic Fungal Infection in CF:*** As the median age of CF patients and the intensity of antibacterial antibiotic treatment increases, multi-drug resistant bacterial organisms and fungi such as *A. fumigatus* have emerged*.* Their prevalence increases with advancing age and increasing severity of lung disease. Whereas the pathophysiological role of the major bacteria, *Pseudomonas aeruginosa* and *Staphylococcus aureus* is well established, the relevance of fungi is poorly understood. Aspergillus fumigatus constitutes the major fungal organism commonly found in lower airway specimens from CF patients. Data on its prevalence vary among centres but larger series have reported a prevalence rate ranging from 21 to 46 % in sputum-producing patients^13-16^. A number of risk factors for *A. fumigatus* infection have been identified: *A. fumigatus* is associated with increasing age (paralleling an increase in severity of lung disease) and appears to be more common in patients living in rural areas^17;18^. It has also been described to be associated with maintenance therapy with oral or inhaled antibiotics as well as long term infection with *P. aeruginosa*, although the latter two factors may be interrelated^19-21^. In addition, a genetic modifier in the promoter region of the IL-10 gene that increases IL-10 production has recently been found to influence the rate of *A. fumigatus* infection^22^.

***Prevalence of Aspergillus infection in Canadian and Australian CF Patients:*** Data from the Canadian Cystic Fibrosis Foundation registry on 3453 CF patients in 2002 indicate that *Aspergillus fumigatus* was cultured from 9% of Canadian CF patients. This is likely an under-estimate of the true overall prevalence of aspergillus infection in Canadian CF patients since: 1) some clinical microbiology laboratories do not routinely culture for fungal pathogens using mycological media and therefore these centers will report no aspergillus growing from their patients’ sputums, and 2) results were only obtained from the first sputum culture done on each patient in 2002. Many patients may be chronically infected with aspergillus, but aspergillus may not be retrieved from every sputum culture taken, because in some instances, bacteria will overgrow the culture plate and make identification of concomitant fungal organisms difficult or impossible. Thus estimation of annual prevalence of infection from a single sputum culture is bound to underestimate the true prevalence of infection with aspergillus.

Data from the Canadian CF patient registry also suggest that the prevalence of aspergillus infection varies amongst regions in Canada. Prevalence was highest in Quebec with 14% of patients culturing aspergillus from their first sputum culture in 2002, and was lowest in the Atlantic provinces where reported prevalence of infection was only 3%. Whether these prevalences reflect true prevalence rates of infection, or whether the apparent low rate of infection in Atlantic Canada reflects failure of local laboratories to culture for fungal pathogens is unclear from the data. Data is also available from the Australian CF Patient Data Registry. In 2003 there were 2350 patients listed in the registry, and 16.4% were culture-positive for *Aspergillus fumigatus*. Prevalence of aspergillus infection was similar in adults (16.2 %) and in pediatric Australian CF patients (16.6 %).

Data from individual CF centers which routinely culture for Aspergillus species is likely more accurate than registry data. Results from the Toronto adult CF clinic indicate that 115 of 326 adult CF patients (35%) had at least 2 sputum cultures positive for Aspergillus fumigatus in 2005. Similar data from the Toronto pediatric CF clinic indicate that 61 of 234 patients (26%) had at least 2 sputum cultures positive for Aspergillus fumigatus in 2005. Prevalence of infection is higher in adults compared to children and this likely reflects an increased exposure to antibiotics in adult patients and associated selective pressures for fungal colonization/infection. It thus appears that the true prevalence of chronic aspergillus infection in Canadians with CF ranges from 26-35%.

***Allergic bronchopulmonary aspergillosis (ABPA) in CF Patients:***

A subgroup of CF patients colonised with *A. fumigatus* develop allergic bronchopulmonary aspergillosis (ABPA), a diagnosis that is based on established clinical and laboratory criteria^23^. The Epidemiologic Study of Cystic Fibrosis studied a series of Canadian and US patients with CF who were > 4 years of age during 1993-96. Of 14,210 North American CF patients who underwent ABPA diagnostic laboratory testing, 281 (2%) received a diagnosis of ABPA. To be diagnosed with ABPA the patient must meet all of the following five CF Consensus Conference criteria:

1) Acute or subacute clinical deterioration not attributable to another etiology.

2) Serum total IgE concentration > 1000 IU/ml (2400 ng/mL).

3) Immediate cutaneous reactivity to Aspergillus (skin test wheal > 3 mm in diameter) or presence of serum IgE antibody to *A. fumigatus*.

4) Precipitating antibodies to *A. fumigatus* or serum IgG antibody to *A. fumigatus.*

5) New or recent abnormalities on chest radiography that have not cleared with antibiotics or physiotherapy.

Our clinical trial will screen potentially eligible patients for ABPA with serum IgE and an IgE RAST serologic test against aspergillus. Itraconazole has not been proven to be of benefit for CF-associated ABPA although there is some open-label trial evidence that addition of itraconazole to systemic steroid therapy in CF patients with ABPA may facilitate a decrease in corticosteroid use. Patients who screen positive for ABPA (serum IgE > 1000 IU/ml and a positive IgE RAST against aspergillus) will be randomized.

***Relevance of A. fumigatus in CF patients:***

The clinical significance of *A. fumigatus* in patients not developing ABPA is largely unknown. Infection with *A. fumigatus* in CF patients may be transient and species genotype can change during the course of the infection^4;24;25^. The serologic response to *A.* fumigatus in CF patients without ABPA has been assessed in multiple studies, and these studies consistently reported elevated IgG and/or IgE antibodies in the majority of patients whose respiratory secretions contain aspergillus organisms^26-33^. The presence of a host antibody response to the organism would support the concept that *A. fumigatus* is not simply colonizing CF airways, but is acting as a relevant pathogen.

A recent study from Israel and the UK suggests that chronic aspergillus infection of the airways, in the absence of ABPA, may pose a significant health risk to CF patients ^34^. The authors describe a group of CF patients with sputum cultures positive for *A. fumigatus* who presented with respiratory deterioration that did not respond to appropriate antimicrobial treatment. Treatment with antifungal agents (itraconazole was used in 5 of 6 patients for 4-24 months) resulted in significant improvement in the patients’ clinical conditions. These data suggest that aspergillus-related bronchitis is an over-looked and largely untreated disease in CF patients. However this hypothesis has not been confirmed by a properly designed prospective clinical investigative study.

**1.4 Relevant Systematic Reviews:**

The current standard of care is to not treat this pathogen in patients who do not have ABPA or invasive fungal disease. **No prospective experimental studies have addressed the question of whether treating aspergillus in patients with cystic fibrosis will improve clinical outcomes.** **This would be the first clinical trial examining this issue.** **Therefore there are no systematic reviews on this subject.** Effective antifungal agents with an acceptable safety profile, such as itraconazole, are now available making it feasible to design an interventional study to study the effect of antifungal therapy on the course of lung disease in CF patients.

***Pulmonary Exacerbations in CF Patients Infected with A. fumigatus:***

Chronic airway infections in patients with CF generally follow a smoldering course punctuated by acute pulmonary exacerbations. Pulmonary exacerbations are characterized by worsening cough, increased sputum production and increased dyspnea^35;36^. Pulmonary exacerbations contribute significantly to the burden of CF disease since they have been shown to be associated with impaired sleep and neurocognitive abilities^37^, impaired quality of life^38^, and with an increased risk of short and longer-term mortality^39;40^.

Treatment of the majority of pulmonary exacerbations requires intravenous antibiotic therapy and provision of chest physiotherapy. The severity of illness usually necessitates hospital admission, although some less severe pulmonary exacerbations can be treated at home with intravenous or oral antibiotic therapy. Hospitalizations for pulmonary exacerbations have been shown to be associated with short-term negative effects on health-related quality of life^38^ and hospitalizations (which usually last 10-14 days) contribute to significant costs to the health care system^11;12^.

Any therapy that can potentially decrease pulmonary exacerbations would be important for patients with CF since this a decrease in exacerbation frequency would be expected to improve quality of life^38^, and decrease both days absent from normal activities and health-care costs. Furthermore exacerbations associated with infections worsen the progression of lung disease in these patients^39^. Accordingly, the frequency of exacerbations is a strong predictor of morbidity and mortality^39^, and a treatment that reduces exacerbations thus has major clinical relevance.

A search of the Toronto adult and pediatric CF patient data registry was undertaken for the year 2005 to identify patients who were chronically infected with aspergillus and to determine exacerbation rates in this cohort. Exacerbation was defined as hospitalization for respiratory symptoms and/or treatment with home intravenous antibiotics. Of the 115 adult CF patients chronically infected with aspergillus (ie. at least 2 positive cultures for aspergillus in 2005), 29% experienced at least one pulmonary exacerbation in the first six months of 2005 requiring IV antibiotics. In contrast, only 14% who did not culture aspergillus from their sputum had a pulmonary exacerbation during the first six months of 2005 (p < 0.01).

Of the 61 pediatric patients from the Toronto clinic who were chronically infected with aspergillus, 23% experienced at least one exacerbation during the first six months of 2005 requiring IV antibiotics. In contrast, only 9% of 142 pediatric patients in Toronto who did not culture aspergillus from their sputum had a pulmonary exacerbation during the first six months of 2005 (p < 0.01). Thus it appears from pediatric and adult data that the risk of pulmonary exacerbation is approximately two to three times higher in Canadian CF patients who are chronically infected with aspergillus compared to those who are not. Chronic infection with aspergillus may be associated with increased risk of exacerbations, either because A. fumigatus is a pathogenic organisms that directly causes exacerbations, or because chronic aspergillus infection is associated with sicker patients who are more predisposed to exacerbations.

Data from the Australian CF Registry is similar to Canadian data. 65.9% of Australian pediatric patients who were chronically infected with aspergillus were hospitalized for a respiratory exacerbation in 2003, compared to a pediatric hospitalization rate of 34.5% in those who were not aspergillus-infected (p < 0.0001). Similarly, 61.7% of Australian adults who were chronically infected with aspergillus were hospitalized for a respiratory exacerbation in 2003, compared to an adult hospitalization rate of 44.0% in those who were not aspergillus-infected (p = 0.04). In total Australians with chronic aspergillus infection had 1.23 (sd 1.44) admissions for IV antibiotics per patient-year in 2003 compared with patients who were not infected with aspergillus who had 0.74 (sd 1.25) admissions per patient-year. This corresponds to a risk ratio of 1.66, indicating that Australian CF patients who were infected with aspergillus were much more likely to require hospital admission for pulmonary exacerbations.

***Itraconazole for Treatment of Aspergillus:*** The first-line treatment for *A. fumigatus* is itraconazole, a triazole antifungal agent with a broad activity spectrum against fungi and a favourable pharmacokinetic and safety profile amongst CF patients. Itraconazole is indicated by Health Canada for treatment of invasive and non-invasive pulmonary aspergillosis in immunocompromised and non-immunocompromised patients. Itraconazole is metabolized by side-chain hydroxylation to 14-hydroxy itraconazole, and Aspergillus fungi are susceptible to both the parent drug and the hydroxylated metabolite^41^. The administration of oral itraconazole 2.5 mg/kg BID in patients with CF achieves steady-state concentrations in a maximum of 8 days^41^. Itraconazole has proven efficacy against *Aspergillus fumigatus* *in vitro* with a low rate of innate resistance. Itraconazole has been used in studies of both CF and non CF patients with ABPA^42^. Once daily oral therapy has proven efficacy for ABPA in non-CF patients and reduction of fungal load has been demonstrated in these studies. A randomized, double-blind trial of treatment with either 200 mg of itraconazole twice daily or placebo for 16 weeks in non-CF patients who had corticosteroid-dependent allergic bronchopulmonary aspergillosis demonstrated significant clinical responses with reductions in corticosteroid dose, serum IgE concentration, and improvements in exercise tolerance and pulmonary-function tests in 13 of 28 patients in the itraconazole group (46 percent), as compared with 5 of 27 patients in the placebo group (19 percent, P=0.04)^43^.

Thus far there are no clinical studies or trials that suggest superiority of other antifungal drugs (such as voriconazole) for the treatment of aspergillus infection in non-immunocompromised patients. Voriconazole is a newer drug, and is now considered the drug of choice for angioinvasive fungal infections in transplant patients and patients with hematologic malignancies. However voriconazole is not indicated for non-invasive fungal disease, and long-term 6 month treatment with voriconazole has not been studied. Furthermore voriconazole is associated with a relatively high incidence of hepatotoxicity and fatal hepatic failure, rash (including fatal Stevens Johnsons syndrome), and retinal disease including color blindness which can occur in up to 25% of treated patients. Given it’s severe toxicities, it is not feasible to use voricanazole for 6 months of chronic therapy in patients who have non-angioinvasive CF associated fungal infection.

Pharmacokinetic data providing a rationale for an itraconazole dosing strategy in CF patients are available from 2 published studies (8.9) as well as from a multi-center pharmacokinetic study performed in Europe^44^. These pharmacokinetic studies in CF patients demonstrated evidence for adequate fungal-killing concentrations of itraconazole in CF sputum ^41;45^. Itraconazole has been found to be safe and well tolerated in these pharmacokinetic studies in CF patients. It is therefore feasible to prospectively assess whether treatment with itraconazole of CF patients who are chronically infected with *A. fumigatus* but who do not have ABPA, will have a positive impact on the course of these patients’ lung disease.

**1.5 How will the results of this trial be used?**

We expect that this study will demonstrate beneficial effects of itraconazole therapy in *A. fumigatus*- infected patients with cystic fibrosis. If confirmed, this should have a positive effect on both health status and long term evolution of lung disease in patients with cystic fibrosis. Results of the study would be disseminated at international meetings, in print form, and dissemination to caregivers would also occur directly through the Canadian and US CF Foundations.

If we do see a positive treatment effect in CF patients, then this would support our hypothesis that *A. fumigatus* is causing disease, and that the organism is not simply an innocent colonizer of diseased airways. *Aspergillus* is also commonly cultured from the respiratory secretions of patients with other chronic lung diseases, such as non-CF bronchiectasis and COPD. A positive result from our clinical trial would be an impetus to conduct future clinical trials to test the hypothesis that treatment of *Aspergillus* colonization in patients with COPD and non-CF bronchiectasis might result in clinical benefits for these other patient populations as well.

**The proposed trial:**

**2.1 Study Design**

The study will be a double-blind, randomized placebo-controlled, multi-centre, clinical trial incorporating two parallel treatment arms.

**2.2 The planned trial intervention**

Patients will be randomly allocated to one of two treatment arms:

1) Daily oral itraconazole for a 24 week treatment period, or

2) Identical placebo given in matched quantities of tablets for a 24 week treatment period.

Dosing of itraconazole has been calculated to provide a daily dose of approximately 5 mg/kg/d as per CF Consensus Guidelines. Itraconazole will be given in tablets containing 100 mg each once daily by mouth, unless the dose exceeds 200 mg/day, in which case it will be given twice daily.

Patients weighing 20 to 34 kg will receive 1 tablet (each tablet containing 100 mg itraconazole or placebo) once daily

Patients weighing 35 to 54 kg will receive 2 tablets (each tablet containing 100 mg itraconazole or placebo) once daily

Patients weighing 55 to 69 kg will receive 3 tablets (each tablet containing 100 mg itraconazole or placebo) divided into 2 doses (2 in the morning and one at night)

Patients weighing 70 kg or more will receive 4 tablets (each tablet containing 100 mg itraconazole or placebo) divided in two equal doses

Itraconazole or placebo will be taken after eating in the morning and in the evening. The drug will be taken with orange juice or 8 oz of a cola beverage (Coca-Cola ^TM^) in order to maximize oral absorption. Absorption of itraconazole can be reduced in patients taking medications which suppress gastric acid production, and patients will be asked to take any prescribed proton pump inhibitors or H2-antagonists at lunchtime to avoid interference with absorption of itraconazole. All study patients will otherwise continue standard therapy for their CF as prescribed by their treating physician.

**2.3 Randomization Method:** Patients will be seen in the CF clinic after they have had their screening bloodwork and they will be reviewed to determine if they meet study eligibility criteria. Those who meet the inclusion criteria will be randomized after informed consent is obtained. The randomization process will consist of a computer-generated random listing of the two treatment allocations blocked by variable blocks of two or four and stratified by site. Randomization will be through central allocation of a randomization schedule and will be coordinated by the Ottawa Health Research Institute. Itraconazole or placebo study medications will be dispensed by the site research pharmacist according to the patient’s randomization assignment.

**2.4 Blinding:**

This study will be double blinded. The placebo medication will be identical in taste and appearance to the itraconazole. Study drugs and placebo will be prepared by the central study pharmacy at the Ottawa Hospital and shipped to participating centres for distribution by the local research pharmacist according to randomization assignment.

**2.5 Patient Selection:**

A higher yield of fungal positive cultures is achieved with use of specific mycological culture media rather than with standard bacteriologic media in the clinical laboratory setting^46^. Therefore we will ask the microbiology laboratories at the hospital sites that participate in our study to routinely use mycologic media for all CF sputum cultures beginning 6-12 months before the start of the trial in order to identify a maximum number of patients with chronic aspergillus infection who will be potentially eligible.

**Inclusion Criteria*:***

- Diagnosis of CF as defined by two or more clinical features of CF and a documented sweat chloride > 60 mEq/L by quantitative pilocarpine iontophoresis test or a genotype showing two well characterized disease causing mutations
- Patient must be known to be chronically colonized with *Aspergillus fumigatus* (at least 2 sputum cultures within the last 12 months which have grown *Aspergillus fumigatus*, one of which must have been obtained within 4 months of randomization).
- Patients must be clinically stable at randomization according to the investigator. In addition, clinical stability will be defined as no use of **new** inhaled, oral or intravenous antibiotics or oral or intravenous corticosteroids during the 14-day period prior to randomization.
- 6 years of age and older at randomization
- Patients must weigh at least 20 kg at the time of randomization
- Patient must be able to perform lung function testing.
- Post-menarche females must be using an effective form of contraception.

**Exclusion criteria:**

- Inability to give informed consent.
- Respiratory culture positive for *B. cepacia* complex within one year of randomization
- Renal function abnormalities - Creatinine > 1.5 times normal within a 30 day period prior to randomization
- Liver function abnormalities - AST or ALT ≥ 2.5 times the upper limit of normal within a 30 day period prior to randomization
- Neutropenia , absolute neutrophil count ≤ 1000 within a 30 day period prior to randomization
- History of biliary cirrhosis or portal hypertension.
- Investigational drug use within 30 days of randomization date.
- History of alcohol, illicit drug or medication abuse within 1 year of screening
- History of lung transplantation or currently on lung transplant list
- Positive serum pregnancy test at screening (to be performed on all post-menarche females)
- Current treatment or previous history of treatment, with oral or intravenous antifungal agents within the last 12 months
- Pregnant or breastfeeding

Itraconazole can potentially interact with the following drugs- patients taking any of these drugs will be excluded from entering the trial: Immunosuppressives: Cyclosporine or Tacrolimus or Sirolimus, Antihistamines: Astemizole or Terfenadine, Hypnotics: Midazolam or Triazolam or Alprazolam, Cisapride , Pimozide, Quinidine, Statins: Atorvastatin, Lovostatin, Simvastatin, Tadalafil, Eletriptan, almotriptan, Vardenafil, Ergot derivatives, HIV antiretroviral agents: Didanosine, Nevirapine, Ritonavir, Indinavir.

**2.6 Proposed duration of the double blind treatment period:** Twenty-four weeks.

*Rationale for duration of treatment:*

Previous trials studying chronic antibiotic therapy for cystic fibrosis, such as the trials of inhaled tobramycin (TOBI) and trials of oral azithromycin therapy, have traditionally been of 24-weeks duration. These studies have been able to demonstrate significant biologic and clinical effects after 24 weeks of chronic antibiotic therapy, and chronic use of these therapies have become standard of care based on these trial results. Based on previous clinical studies we would expect eradication of fungal infection, or at least suppression of growth and a reduction of fungal burden, within several months of beginning therapy with itraconazole. In this context, a treatment period of 24 weeks with itraconazole should show a significant microbiological effect and an effect on pulmonary exacerbations and lung function. A 24-week period is also necessary in order to accumulate enough pulmonary exacerbation events to be able to show a difference between the two treatment groups.

**2.7 Study Period, frequency and duration of follow-up:** The multi-centre study will recruit patients over a period of 18 months and follow each recruited patient for a total of 48 weeks after they are randomized. Patients will be followed for 24 weeks while on study therapy and for an additional 24 weeks after study therapy has been discontinued. Primary outcomes for the study will be determined at 24 weeks, at the time point where the patient has just completed 24 weeks of active study therapy. However, we will follow patients for an additional 24 weeks after study therapy has been discontinued (ie. from weeks 24 to week 48 post-randomization) to determine if there are any lasting positive or negative carry-over effects of therapy with itraconazole. Efficacy and safety outcomes measured at 48 weeks will be assessed as secondary outcomes.

**2.8 Outcome Measures**

***Primary outcome measure:***  The primary outcome measure will be the proportion of patients who experience a respiratory exacerbation requiring intravenous antibiotics in the two treatment groups over the 24 week trial treatment period.

A pulmonary exacerbation will be defined according to clinical criteria published by the 1994 Cystic Fibrosis Foundation Microbiology and Infectious Disease Consensus Conference^24^. We have successfully used identical exacerbation criteria as an outcome in our previous multi-center CF clinical trial^47^. An exacerbation will be defined as the presence of at least three of the following 11 new findings or changes in clinical status when compared to the most recent baseline visit^24^:

- Increased cough
- Increased sputum production and/or change in appearance of expectorated sputum
- Fever (>38 ° C for at least four hours in a 24-hour period) on more than one occasion in the previous week
- Weight loss > 1 kg or 5% of body weight associated with anorexia and decreased dietary intake
- School or work absenteeism (due to illness) in the previous week
- Increased respiratory rate and/or work of breathing
- New finding on chest examination (eg. rales, wheezing, crackles)
- Decreased exercise tolerance
- Decrease in FEV_1_ of >10% from previous baseline study within past three months
- Decrease in hemoglobin saturation (as measured by oximetry) from baseline value within past three months of >10%
- New finding(s) on chest radiograph

An exacerbation must meet the above definition and it must also be associated with an acute change in regular CF medications, defined as physician-directed acute use of new intravenous antibiotics. The study will monitor patients for exacerbation via monthly telephone calls and via patient visits at 4, 12, 24, and 48 weeks after randomization.

CF pulmonary exacerbation was chosen as the primary outcome variable since it represents a well-defined clinical outcome that is understandable to patients and their physicians. Studies suggest that CF pulmonary exacerbations negatively affect health-related quality of life^38^. Furthermore, pulmonary exacerbations are associated with impaired sleep and neurocognitive abilities^37^ and with increased patient mortality^39;40^. This endpoint is also an important economic outcome, since those patients who have respiratory exacerbations generate higher health-care costs, both for physician visits, hospitalisations, and drug costs^11;12^.

We deliberately chose respiratory exacerbation requiring **intravenous antibiotics** as our primary outcome, rather than respiratory exacerbations requiring **oral or intravenous** antibiotics. The trial protocol will require patients to take oral itraconazole for 24 weeks and we did not feel that clinicians or patients would adopt this intervention in the future unless our clinical trial could show that the incidence of moderate and severe pulmonary exacerbations was reduced. Reduction of mild pulmonary exacerbations requiring brief courses of oral antibacterial antibiotics was not felt to be important enough to justify 24 weeks of therapy with itraconazole.

Adjudication of the primary outcome

For each suspected patient exacerbation a full report describing the circumstances of the suspected exacerbation will be prepared by study personnel. For every suspected exacerbation reported by the patient, we will obtain copies of the written medical record of the patient encounter. The assembled data from this visit will be presented to a blinded study Adjudication Committee for review.

**2.9 How will the outcome measures be measured at follow-up?**

***Secondary outcomes that will be assessed will include:***

1) The proportion of patients who experience a respiratory exacerbation requiring **intravenous** **or oral antibiotics** over the 24 week trial treatment period.

2) Absolute and relative changes in the forced expiratory volume in one second (FEV_1_) and forced vital capacity (FVC) over the 24-week treatment period.

3) Changes in disease-specific health-related quality of life over the 24 week study treatment

period as assessed by the Cystic Fibrosis Questionnaire (CFQ-R)- a disease-specific instrument that measures health-related quality of life for adolescents aged 14-18 and for adults with cystic fibrosis. The CFQ-R has been demonstrated in the European and American CF population to be reliable, valid, and responsive to change, and to correlate with SF-36 scores, pulmonary function, and disease severity. Scores are standardized on a 0 to 100 point scale with higher scores representing better quality of life. For children 6-14 years of age we will use the Cystic Fibrosis Children Questionnaire (CFQ-UK Children), a modified version of the CFQ-R that has been adapted and validated for use in the pediatric age group.

4) No. of exacerbations that result in hospitalisation over the 24-week treatment period.

5) Total number of hospitalisations (all cause) over the 24-week treatment period.

6) The mean number of exacerbations per patient over the 24 –week period.

7) The time to first exacerbation.

8) Premature discontinuation of study medication, for reasons of adverse effects or lack of

efficacy, as judged by the patient’s physician.

9) Incidence of adverse effects and serious adverse events.

10) Eradication of fungal infection/colonization. Those patients whose sputum is negative for fungal organisms on all 3 occasions at 24, 36 and 48 weeks after randomization will be considered to have had their sputum eradicated of fungus.

All efficacy and safety outcomes listed above will be assessed at 24 weeks, but also at 48 weeks after randomization, to determine if there are any lasting positive or negative carry-over effects of 24 weeks of therapy with itraconazole. Quality of life and pulmonary function will be measured at the time of randomization, and thereafter at 4, 12, 24, and 48 weeks after randomization.

**2.10 Sample Size Calculations:**

The primary outcome measure will be the proportion of patients who experience a respiratory exacerbation requiring intravenous antibiotics in the two treatment groups over the 24 week trial observation period. In our combination antibiotic susceptibility trial we enrolled and followed 251 patients with cystic fibrosis and randomized patients when they experienced a pulmonary exacerbation. Eighty-seven patients (35%) experienced an exacerbation requiring intravenous antibiotics within 24 weeks of enrolment^47^. Similarly, 32% of placebo-treated patients in The Australian hypertonic saline trial experienced a CF exacerbation requiring intravenous antibiotics within 24 weeks^48^. Finally, in the TOBI study 100 of 258 actively-treated patients (39%), and 135 of 262 placebo-treated patients (52%) experienced at least one pulmonary exacerbation requiring intravenous antibiotics over the 24-week trial^49^.

Additionally, data from the 2005 Toronto CF Data Registry suggests that of Canadian adult and pediatric patients who are chronically infected with aspergillus, 27% experienced at least one respiratory exacerbation during the first six months of 2005 which required hospital admission and/or home intravenous antibiotic therapy. According to the statistician (Dr. Mary Corey) who administers the database this is likely an under-estimation of the true rate of exacerbations, since data on hospitalizations or home IV therapy can be missing or incomplete within the database.

Thus, based on data from the above-mentioned three recently completed studies, and based on the Toronto database data, we conservatively expect that 34% of placebo-treated patients in our trial will experience a pulmonary exacerbation requiring intravenous antibiotics over the 24 week duration of our trial. The Trial Investigators (4 Canadian CF physicians and 4 Australian CF physicians) determined that a 15% absolute difference in 24-week exacerbation rates between the two treatment groups would be the minimal clinically significant difference that would be important for the study to detect.

An MCID of 15% was decided upon for two reasons: 1) Data from Toronto suggests that at least 27% of pediatric and adult patients who are chronically infected with *A. fumigatus* experience a pulmonary exacerbation requiring IV antibiotics within a 24-week period, and that this risk is reduced to 11% in those who are not infected with Aspergillus (ie. a risk reduction of 16%). Thus a 15% absolute risk reduction might be expected if we can eliminate aspergillus infection in those study patients who are randomized to active anti-fungal treatment. 2) The study intervention is lengthy (24 weeks), expensive (approximately $16.00 per day for adults), and not without risk of side-effects. Therefore it was argued that unless a relatively large signal is seen, ie. unless the absolute risk reduction for exacerbation is at least 15%, then widespread implementation of the intervention would not be justified in the larger CF population. Assuming a binomial distribution for exacerbation rates, with a 15% absolute risk reduction between itraconazole vs. placebo, a two-sided alpha error of 0.05, and a beta error of 0.20, 148 patients per arm or 296 subjects in total are required. In order to allow for a 5% non-compliance rate (Lachin formula), a total sample size of 328 subjects is required.

**2.11 Feasibility of Recruitment**

The study will recruit patients 328 patients over a period of 18 months from 18 Canadian and Australian pediatric and adult CF clinics. The ten participating Canadian clinics have a total clinic population of approximately 2000 patients, and the eight participating Australian clinics have a total clinic population of approximately 1500 patients. Given that at least 25-30% of the CF patient population is expected to be chronically infected with aspergillus, this would leave us with a pool of 875 Canadian and Australian patients to draw upon in order to randomize our expected sample size of 328 patients. Thus recruitment and randomization of eligible patients should be feasible. Retention of patients in the trial is not expected to be problematic since patients return to the same local CF clinic for all of their care. Our previous Canadian-Australian CF trial randomized 132 patients, and none were lost to follow-up.

**2.12/2.13 Compliance and Losses to Follow-up**

The patient’s treating physicians will be instructed not to prescribe anti-fungal antibiotics to enrolled study patients. However, if a patient’s physician insists that the patient must be started on an anti-fungal agent during the study period, then the study medication will be stopped (to avoid potential over-dosage). The patient will continue to be followed for the duration of the study period and results will be analysed according to the intention-to-treat principle. Premature discontinuation of study medication, for reasons of adverse effects or lack of efficacy, will be assessed as a secondary outcome.

Based on previous trials, we expect that 5-10% of participants will prematurely discontinue study medication but continue in the study, and that less than 5% of patients will withdraw from the study and be lost to follow-up. Losses to follow-up are rare in CF trials since patients are all followed in a central CF clinic. For instance, in our combination antibiotic susceptibility trial there were no losses to follow-up despite a 4.5 year study duration^47^. Patient compliance will be assessed by assessing serum itraconazole levels at 12 and 24 weeks. Patients who have detectable blood levels of itraconazole at 12 and 24 weeks will be judged to have been compliant. Patients who are non-compliant will still be included in the primary intention-to-treat analysis.

**2.14 Statistical Analysis**

*Principal Analysis of the Primary Outcome Measure:*

The final analysis will be performed with an ‘intention to treat’ approach. Patients will be included in the analysis according to the group to which they were randomized regardless of cross-over or compliance. Baseline characteristics of patients in the two treatment arms will be assessed using frequency distributions and univariate descriptive statistics including measures of central tendency and dispersion.

The principal analysis of the exacerbation proportions in the two treatment groups will be conducted using an unadjusted Fishers exact test comparing the proportion of events in the two treatment groups. A logistic regression procedure will be employed to adjust raw exacerbation proportions using important covariates that may be unbalanced at baseline that may influence outcomes (such as gender, baseline FEV_1_, home oxygen use, use of chronic inhaled antibiotics, use of chronic oral antibiotics, use of DNAse, use of hypertonic saline, and co-morbid illnesses).

We chose the proportion of patients having at least one exacerbation as a primary outcome measure rather than survival time to exacerbation. We believe that it is more clinically relevant to both patients and physicians to know whether or not a patient exacerbates rather than when they exacerbate. Therefore, an analysis of raw proportions followed by logistic regression procedures were chosen over log-rank tests followed by Cox proportional hazards modelling. Both alternative analytic approaches mentioned will be performed and reported.

*Principal analysis of the secondary outcome measures:* All secondary outcome measures will be analyzed using an ‘intention to treat’ analysis. Any patients who drop out of the study before the 24 week mark will have measurements of airflow obstruction, dyspnea, and quality-of-life done on the last study visit carried forward for purposes of the analysis (ie. endpoint analysis, with the last observation carried forward). Continuous outcome measures including absolute and percent changes in FEV_1_ and changes in the scores of the components and the total score of the Cystic Fibrosis Questionnaire (CFQ-R) will be analysed using multi-variate repeated measures analysis of variance using the SAS Proc Mixed program. This procedure allows us to model the covariance structure of the data and allows for missing observations. Kaplan-Meier survival curves will be used to describe the probability of remaining exacerbation-free in the two treatment arms as a function of time from randomization into the study. The curves will be compared using the log-rank test statistic. Subsequently, Cox proportional hazards modeling will be performed to adjust for other variables that are unbalanced at baseline (such as gender, baseline FEV_1_, home oxygen use, use of chronic inhaled antibiotics, use of chronic oral antibiotics, use of DNAse, use of hypertonic saline, and co-morbid illnesses) that could potentially affect the time to next exacerbation. The mean number of exacerbations/patient will be calculated using a weighted approach to account for each patient’s duration of follow-up in the trial. The 95% confidence interval and the P value of the rate ratio will be obtained by using Poisson regression analysis models with log transformations of the number of exacerbations and the duration of follow-up.

***Other Considerations:*** P-values will be reported as two-sided. Data will be displayed as proportions and means with 95% confidence intervals or medians with interquartile ranges as appropriate. Likewise, measures of effect, odds ratios (logistic regression) and hazards ratio (Cox proportional hazards) will be presented with 95% confidence intervals.

**2.15 Planned Subgroup Analyses:** Using the approach outlined for primary and secondary analyses, we will perform three subgroup analyses for: 1) Patients with baseline FEV_1_ less than 50% of predicted; 2) Patients who screen positive for ABPA (serum IgE > 1000 IU/ml and a positive IgE RAST against aspergillus); 3) Patients < 18 years of age. These analyses will primarily be hypothesis-generating in nature.

**2.16 Interim Analysis:** An independent data safety monitoring board will be appointed and the DSMB will oversee the trial, monitor adverse events, and perform an interim analysis. One interim analysis will be performed once 50% of patients have been accrued to determine if the experimental therapy is beneficial or hazardous. O’Brien-Fleming group sequential stopping rules were chosen to maintain an overall significance level of 0.05. Significance boundaries will be symmetrical with alpha = 0.005 for the interim analysis and alpha = 0.048 for the final analysis.

**2.17 Economic Issues:** A pharmaco-economic analysis evaluating the costs of this therapy will also be performed (see appendix for details). The objectives of the economic analysis will be to 1) estimate the cost of treating a CF exacerbation for the two proposed treatment strategies, 2) perform a cost minimization analysis by comparing the costs for the treatment strategies, and 3) conduct a cost-effectiveness analysis. The cost-effectiveness analysis will be assessed in terms of the cost per life year gained and QALY gained; and the analysis will be conducted from a health care system perspective. The cost assessment of treatment strategies will include only physician/clinic visit costs, hospitalization costs and drug costs since these are assumed to be the major cost drivers for treatment of CF exacerbation.

**2.18 Safety:** Itraconazole is rarely associated with liver function abnormalities and very rarely the drug can cause clinical hepatitis (3.2/100,000 prescriptions). Other potential rare side effects are hypokalemia and renal function abnormalities (occurring in less than 3% of patients). Patients will have serum electrolytes, liver function tests, and creatinine monitored regularly at 4 weeks, 12, and 24 weeks after randomization. Patients whose liver function tests increase to greater than three times above normal values, or whose creatinine increases to greater than 1.5 times baseline values, or who develop severe hypokalemia (serum potassium < 3.0) will be taken off of the study drug for the duration of the study. Patients with mild hypokalemia (serum potassium of 3.0-3.5) will be prescribed a potassium supplement and electrolytes will be monitored regularly. Further details of study monitoring for adverse events, and specific protocols and study procedures for monitoring of potential drug interactions of itraconazole with anticonvulsants, anticoagulants, oral hypoglycaemic agents, systemic and inhaled steroids, and digoxin can be found in the study appendices.

**2.19 Estimated Cost and Duration of the Trial:** 3 years; $2,257,038.00

**Details of The Trial Team:**

**3.1 Day to Day Management of the Trial:**

The trial project manager will be Katherine Vandemheen (BScN) who has extensive expertise coordinating and managing large multi-center clinical trials in respiratory and emergency medicine. The Coordinating Centre will be located at the Ottawa Health Research Institute, Clinical Epidemiology Unit (CEU). The Ottawa CEU has considerable experience in managing all aspects of multi-center clinical trials and is currently overseeing the development, implementation and management of over 30 clinical trials.

The co-principal investigators (Drs. Felix Ratjen and Shawn Aaron) have extensive training and experience in the conduct of multi-center clinical trials. Dr. Ratjen is one of the world’s leading CF researchers and has published review articles and editorials on CF in The Lancet and in the NEJM. He has first-authored results of CF clinical trials in The Lancet and The American Journal of Respiratory and Critical Care Medicine. Dr. Aaron recently successfully completed a multi-center Canadian-Australian CF clinical trial that was published in the Lancet. In total he has completed three CIHR-sponsored multi-center clinical trials in the areas of cystic fibrosis and COPD and he has first-authored results of these trials in The NEJM , The Lancet, and The Annals of Internal Medicine. Drs. Ratjen and Aaron will serve as the study chairs and will have overall responsibility for the project. The co-applicants and members of the Canadian Trial Executive Committee (Katherine Vandemheen, Bill Cameron, and Dean Fergusson) are established clinician/scientists in CF and respiratory medicine.

**3.2 International Collaboration:**

Canadian and Australian CF clinics will participate. In this way we will have an adequate pool of CF patients infected with aspergillus to draw upon in order to guarantee feasibility of recruitment. We successfully completed a multi-center Canadian/Australian CF clinical trial evaluating combination antibiotic susceptibility tests in 2005^47^, and we are currently conducting another multi-center Canadian/Australian clinical trial of a decision aid for CF patients facing lung transplantation. Thus the infrastructure necessary to run the trial described in this application is already well-developed and fully functioning in Canada and Australia.

**3.3 Proposed Participating Centers:**

Adult and pediatric CF clinics will be invited to participate from: Halifax, Montreal, Ottawa, Toronto, London, Hamilton, Kingston, Calgary, Edmonton, and Vancouver. Australian sites will include: Sydney, Westmead, Brisbane, Newcastle, Melbourne, and Perth. The Australian trial steering committee is composed of: Drs. Peter Wark (Newcastle adult CF clinic), Dr. Peter Bye (Sydney adult CF clinic), Hiran Selvadurai (Westmead peds), Adam Jaffe (Sydney peds), and Scott Bell (Brisbane adult). All of these sites have participated in our previous trials.

**4.0 Other Funding Sources:**

We applied to the Canadian CF Foundation and the Physician Services Foundation for funding for $450,000 to recruit the first 60 study patients from Ottawa and Toronto. The applications were successful and we were awarded $342,800 in total. This money will be used to recruit the first 50-60 patients.

Reference List

1. Ratjen F, Doring G. Cystic Fibrosis. Lancet 2003;361:681-89.

2. Gibson RL, Ramsey BW. Pathophysiology and management of pulmonary infections in cystic fibrosis. Am J Respir Crit Care Med 2003;168:918-51.

3. FitzSimmons SC. The changing epidemiology of Cystic Fibrosis. J Pediatr 1993;122:1-9.

4. Haase G, Skopnik H, Groten T, Kusenbach G, Posselt HG. Long-term fungal cultures from sputum of patients with cystic fibrosis. Mycoses 1991;34:373-76.

5. Dodge JA, Morison S, Lewis PA, Coles EC, Geddes D, Russell G *et al.* Incidence, population, and survival of cystic fibrosis in the UK, 1968-95. UK Cystic Fibrosis Survey Management Committee. Arch Dis Child 1997;77:493-96.

6. Corey M. Canadian Cystic Fibrosis Patient Registry. Canadian Cystic Fibrosis Foundation 1999.

7. Corey M, Farewell V. Determinants of mortality from cystic fibrosis in Canada, 1970-1989. Am J of Epidemiology 1996;143:1007-17.

8. Welch DF, Muszynski MJ, Pai CH, Marcon MJ, Hribar MM, Gilligan PH *et al.* Selective and differential medium for recovery of Pseudomonas cepacia from the respiratory tracts of patients with cystic fibrosis. J Clin Microb 1987;25:1730-34.

9. Robson M, Abbott J, Webb K, Dodd M, Walsworth-Bell J. A cost description of an adult cystic fibrosis unit and cost analysis of different categories of patients. Thorax 1992;47:684-89.

10. Johnson JA, Connolly MA, Jacobs P. Cost of care for individuals with cystic fibrosis in Alberta: a regression approach to determining important cost drivers. Int Arch Allergy Immunol 1996;99:102.

11. Johnson JA, Connolly MA, Jacobs P, Montgomery M, Brown NE, Zuberbuhler P. Cost of care for individuals with cystic fibrosis: a regression approach to determining the impact of recombinant human DNase. Pharmacotherapy 1999;19:1159-66.

12. Lieu TA, Ray GT, Farmer G, Shay GF. The cost of medical care for patients with cystic fibrosis in a health maintenance organization. Pediatrics 1999;103:e72.

13. Schonheyder H, Jensen T, Hoiby N, Andersen P, Koch C. Frequency of Aspergillus fumigatus isolates and antibodies to aspergillus antigens in cystic fibrosis. Acta Pathol Microbiol Immunol Scand 1985;93:112.

14. Burns JL, Emerson J, Stapp JR, Yim DL, Krzewinski, J. *et al.* Microbiology of sputum from patients at cystic fibrosis centers in the United States. Clin Infect Dis 1998;27:158-63.

15. Cimon B, Symoens F, Chabasse D, Nolard N, Defontaine A, Bouchara JP. Molecular epidemiology of airway colonisation by Aspergillus fumigatus in cystic fibrosis patients. J Med Microbiol 2001;50:347-74.

16. Bakare N, Rickerts V, Bargon J, Just-Nubling G. Prevalence of Aspergillus fumigatus and other fungal species in the sputum of adult patients with cystic fibrosis. Mycoses 2003;46:19-23.

17. Skov M, McKay K, Koch C, Cooper PJ. Prevalence of allergic bronchopulmonary aspergillosis in cystic fibrosis in an area with a high frequency of atopy. Respir Med 2005;887-93.

18. Simmonds EJ, Littlewood JM, Hopwood V, Evans EG. Aspergillus fumigatus colonisation and population density of place of residence in cystic fibrosis. Arch Dis Child 1994;70:139-40.

19. Burns JL, Van Dalfsen JM, Shawar RM, Otto KL, Garber RL, Quan JM *et al.* Effect of chronic intermittent administration of inhaled tobramycin on respiratory microbial flora in patients with cystic fibrosis. J Infect Dis 1999;179:1190-96.

20. Bargon J, Dauletbaev N, Kohler B, Wolf M, Posselt HG, Wagner TO. Prophylactic antibiotic therapy is associated with an increased prevalence of Aspergillus colonization in adult cystic fibrosis patients. Respir Med 1999;93:838.

21. Ritz N, Ammann RA. Risk factors for allergic bronchopulmonary aspergillosis and sensitisation to Aspergillus fumigatus in patients with cystic fibrosis. Eur J Pediatr 2005;164:577-82.

22. Brouard J, Knauer N, Boelle PY, Corvol H, Henrion-Caude A, Flamant C *et al.* Influence of interleukin-10 on Aspergillus fumigatus infection in patients with cystic fibrosis. J Infect Dis 2005;191:1988-91.

23. Stevens DA, Moss RB, Kurup VP, Knutsen AP, Greenberger P, Judson MA *et al.* Allergic bronchopulmonary aspergillosis in cystic fibrosis--state of the art: Cystic Fibrosis Foundation Consensus Conference. Clin Infect Dis 2003;37:S225-S264.

24. Neuveglise C, Sarfati J, Debeaupuis JP, Vu Thien H, Just J, Tournier G *et al.* Longitudinal study of Aspergillus fumigatus strains isolated from cystic fibrosis patients. Eur J Clin Microbiol Infect Dis 1997;16:747-50.

25. Rath PM, Ratjen F, Ansorg R. Genetic diversity among isolates of Aspergillus fumigatus in patients with cystic fibrosis. Zentralbl Bakterio 1997;285:450-55.

26. Schonheyder H, Jensen T, Hoiby N, Koch C. Clinical and serological survey of pulmonary aspergillosis in patients with cystic fibrosis. Int Arch Allergy Immunol 1988;85:472-77.

27. Zeaske R, Bruns WT, Fink JN, Greenberger PA, Colby H, Liotta JL *et al.* Immune responses to Aspergillus in cystic fibrosis. J Allergy Clin Immunol 1988;82:73-77.

28. el-Dahr JM, Fink R, Selden R, Arruda LK, Platts-Mills TA, Heymann PW. Development of immune responses to Aspergillus at an early age in children with cystic fibrosis. Am J Respir Crit Care Med 1994;150:1513-18.

29. Murali PS, Pathial K, Saff RH, Splaingard ML, Atluru D, Kurup VP *et al.* Immune responses to Aspergillus fumigatus and Pseudomonas aeruginosa antigens in cystic fibrosis and allergic bronchopulmonary aspergillosis. Chest 1994;106:513-19.

30. Arruda LK, Muir A, Vailes LD, Selden RF, Platts-Mills TA, Chapman MD. Antibody responses to Aspergillus fumigatus allergens in patients with cystic fibrosis. Int Arch Allergy Immunol 1995;107:410-11.

31. Hutcheson PS, Knutsen AP, Rejent.A.J., Slavin RG. A 12-year longitudinal study of Aspergillus sensitivity in patients with cystic fibrosis. Chest 1996;363-66.

32. Wojnarowski C, Eichler I, Gartner C, Gotz M, Renner S, Koller DY *et al.* Sensitization to Aspergillus fumigatus and lung function in children with cystic fibrosis. .Am J Respir Crit Care Med 1997;155:1902-07.

33. Maiz L, Cuevas M, Quirce S, Canon JF, Pacheco A, Sousa A *et al.* Serologic IgE immune responses against Aspergillus fumigatus and Candida albicans in patients with cystic fibrosis. Chest 2002;121:782-88.

34. Shoseyov D, Brownlee KG, Conway S.P., Kerem E. Aspergillus Bronchitis in Cystic Fibrosis. Chest 2006;130:222-26.

35. Cystic Fibrosis Foundation. Microbiology and infectious disease in cystic fibrosis. V[Section 1]. Canadian Cystic Fibrosis Foundation , 1-26. 1994.

36. Rosenfeld M, Emerson J, Williams-Warren J, Pepe M, Smith A, Montgomery AB *et al.* Defining a pulmonary exacerbation in cystic fibrosis. J Pediatr 2001;139:359-65.

37. Dobbin CJ, Bartlett K, Melehan K, Grunstein RR, Bye PT. The effect of infective exacerbations on sleep and neurobehavioral function in cystic fibrosis. Am J Respir Crit Care Med 2005;172:99-104.

38. Britto MT, Kotagal UR, Hornung RW, Atherton HD, Tsevat J, Wilmott JW. Impact of recent pulmonary exacerbations on quality of life in patients with cystic fibrosis. Chest 2002;121:64-72.

39. Liou TG, Adler FR, FitzSimmons SC, Cahill JR, Hibbs JR, Marshall BC. Predictive 5-year survivorship model of cystic fibrosis. Am J of Epidemiology 2001;153:345-52.

40. Mayer-Hamblett N, Rosenfeld M, Emerson J, Goss CH, Aitken ML. Developing cystic fibrosis lung transplant referral criteria using predictors of 2-year mortality. Am J Respir Crit Care Med 2002;166:1550-55.

41. Conway SP, Etherington C, Peckham DG, Brownlee KG, Whitehead A, Cunliffe H. Pharmacokinetics and safety of itraconazole in patients with cystic fibrosis. J Antimicrob Chemother 2004;53:841-47.

42. Elphick H, Southern K. Antifungal therapies for allergic bronchopulmonary aspergillosis in people with cystic fibrosis. Cochrane Database Syst Rev 2000;4:CD002204.

43. Stevens DA, Schwartz HJ, Lee JY, Moskovitz BL, Jerome DC, Catanzaro A *et al.* A randomized trial of itraconazole in allergic bronchopulmonary aspergillosis. N Engl J Med 2000;342:756-62.

44. Huttegger, I. 2006. Personal Communication

45. Sermet-Gaudelus I, Lesne-Hulin A, Lenoir G, Singlas E, Berche P, Hennequin C. Sputum itraconazole concentrations in cystic fibrosis patients. Antimicrob Agents Chemother 2001;45:1937-38.

46. Horvath JA, Dummer S. The Use of Respiratory-Tract Cultures in the Diagnosis of Invasive Pulmonary Aspergillosis. Am J Med 1996;100:171-78.

47. Aaron SD, Vandemheen KL, Ferris W, Fergusson D, Tullis E, Haase D *et al.* Combination antibiotic susceptibility testing to treat exacerbations of cystic fibrosis associated with multiresistant bacteria: a randomised, double-blind, controlled clinical trial. Lancet 2005;366:463-71.

48. Elkins MR, Robinson M, Rose BR, Harbour C, Moriarty CP, Marks GB *et al.* A Controlled Trial of Long-Term Inhaled Hypertonic Saline in Patients with Cystic Fibrosis. N Engl J Med 2006;354:229-40.

49. Ramsey BW, Pepe MS, Quan JM, Otto KL, Montgomery AB, Williams-Warren J *et al.* N Engl J Med 1999;340:23-30.
